# Supplementary material for: Snail promotes ovarian cancer progression by recruiting myeloid-derived suppressor cells via CXCR2 ligand upregulation
Source: Nat Commun. 2018 Apr 27;9:1685. doi: 10.1038/s41467-018-03966-7 (PMC5923228; doi:10.1038/s41467-018-03966-7)
Supplement: Supplementary file 1 — Supplementary Information [file 41467_2018_3966_MOESM1_ESM.pdf]

Supplementary Information for

Snail induces migration of tumor-infiltrating myeloid-derived suppressor cells through CXCR2 ligand upregulation in ovarian cancer

Taki et al.

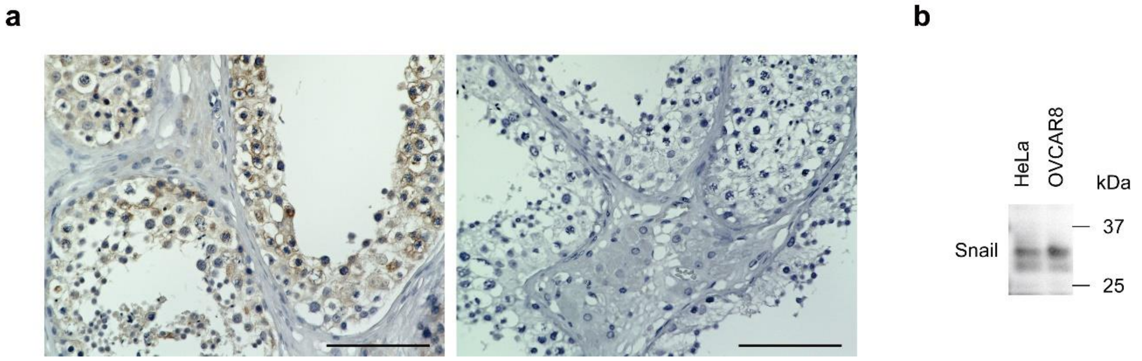

Supplementary Figure 1. Immunohistochemical analysis of anti-Snail antibody in human ovarian cancer samples.

(a) Representative microscopic images of Snail-immunostained human testis (left) and control IgG-immunostained human testis (right). Scale bars, 100 μm. **(b)** Western blot analysis of HeLa (positive control) and OVCAR8 ovarian cancer cell line stained with anti-Snail antibody.

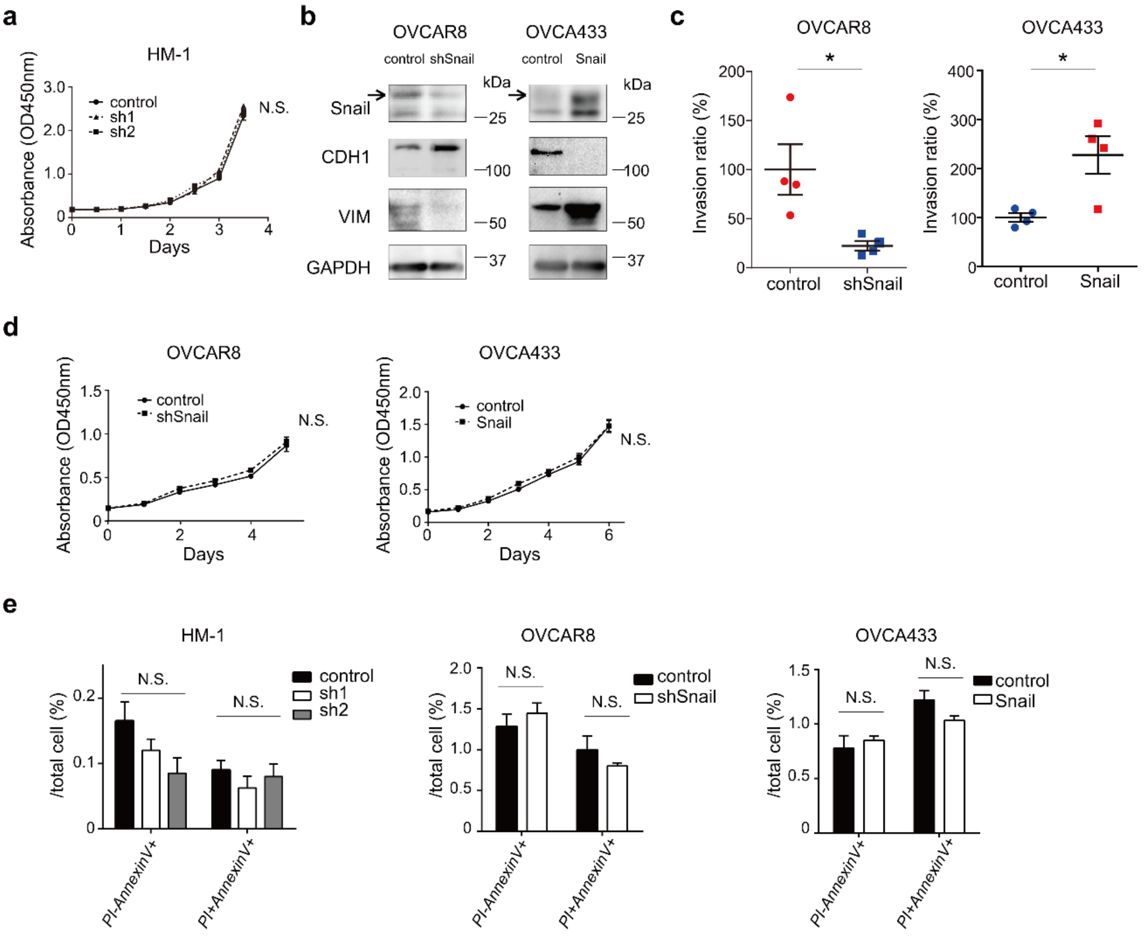

Supplementary Figure 2. Snail induces epithelial-to-mesenchymal transition characteristics in human ovarian cancer cell lines.

(a) Cell proliferation assay for HM-1-control and HM-1-shSnail cells. Mean ± SEM; n = 6; N.S.; not significant (at day 3.5) by unpaired *t* test. **(b)** Western blot analysis of OVCAR8-control, OVCAR8-shSnail, OVCA433-control, and OVCA433-Snail cell lines. CDH1: E-Cadherin, VIM: vimentin. **(c)** Invasion assay for OVCAR8-control and OVCAR8-shSnail (left) cells, and for OVCA433-control and OVCA433-Snail (right) cells. Mean ± SEM; n = 4; *P < 0.05 by unpaired *t* test. **(d)** Cell proliferation assay for OVCAR8-control and OVCAR8-shSnail (left) cells, and for OVCA433-control and OVCA433-Snail (right) cells. Mean ± SEM; n = 6; N.S. (OVCAR8; at day5, OVCA433; at day 6) by unpaired *t* test. **(e)** Apoptosis assay for HM-1 (left), OVCAR8 (middle) and OVCA433 (right) cell lines. Mean ± SEM; n = 4; N.S. by one-way ANOVA with Tukey's multiple comparisons test (HM-1) or unpaired *t* test (OVCAR8 and OVCA433).

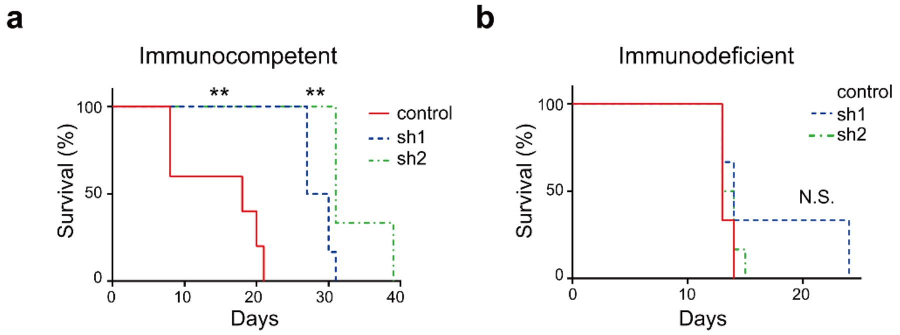

Supplementary Figure 3. Snail inhibits tumor growth in intraperitoneally inoculated immunocompetent mice model.

(a) Survival curves for HM-1-control and HM-1-shSnail intraperitoneally inoculated immunocompetent mice; n = 6. **(b)** Survival curves for HM-1-control and HM-1-shSnail intraperitoneally inoculated immunodeficient mice; n = 6; No significant differences were observed between groups. **P < 0.01 (log-rank test in **a, b**). Averaged data are presented as the mean ± SEM.

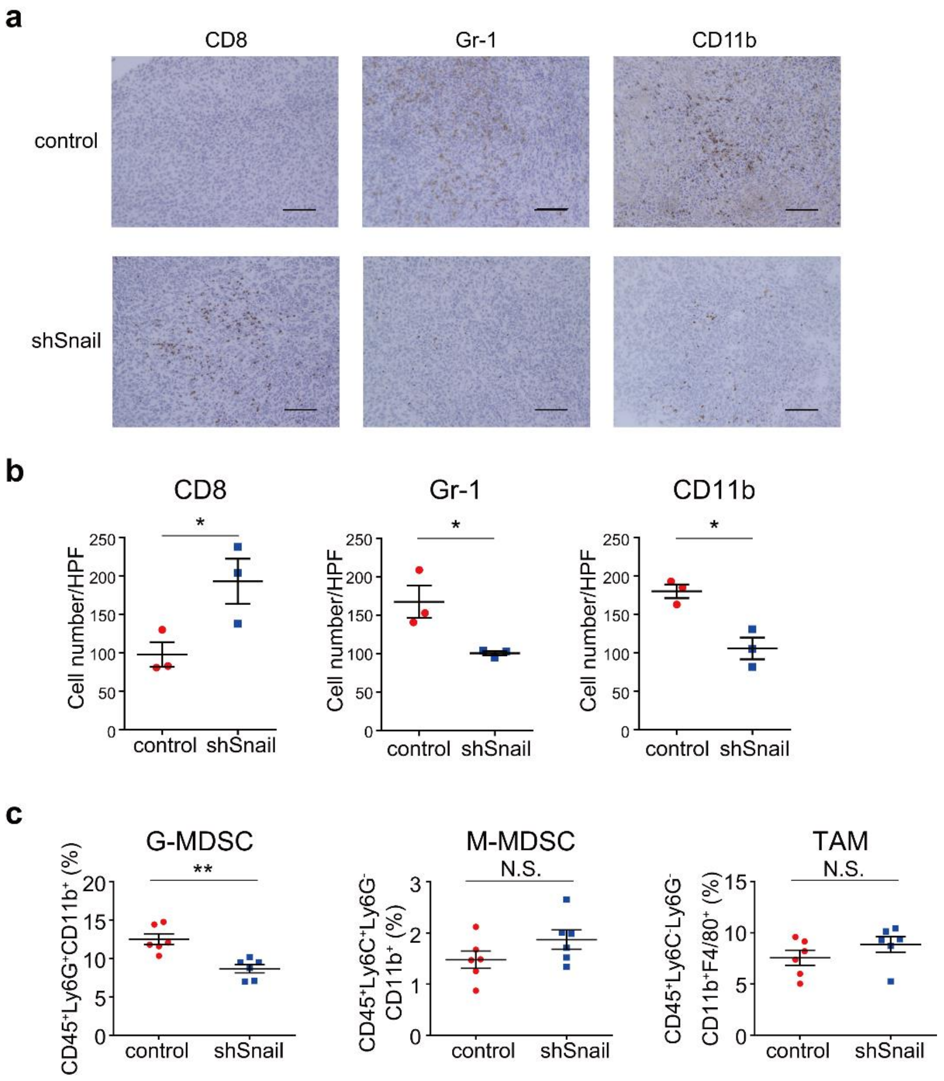

Supplementary Figure 4. Snail induces MDSCs infiltration in mouse tumors.

(a) Representative microscopic images of immunostained HM-1-control (upper panels) and HM-1-shSnail (lower panels) tumors in immunocompetent mice. Scale bar, 100 μm. CD8$^+$ (left), Gr-1$^+$ (middle) and CD11b$^+$ (right). **(b)** Immunostained cell count in HM-1-control and HM-1-shSnail (sh1) intraperitoneal tumors from immunocompetent mice. HPF, high power field; n = 3. CD8$^+$ (left), Gr-1$^+$ (middle) and CD11b$^+$ (right). *P < 0.05. Unpaired *t* test. **(c)** Flow cytometric analysis of subcutaneous HM-1-control and HM-1-shSnail tumors from immunocompetent mice. Percentage of positive cells to total cells is plotted. G-MDSC (left; CD45$^+$Ly-6G$^+$CD11b$^+$; **P < 0.01, n = 6), M-MDSC (middle; CD45$^+$Ly-6c$^+$Ly6G$^-$CD11b$^+$; N.S. n = 6) and tumor-associated macrophages (TAMs) (right; CD45$^+$Ly-6c$^-$Ly6G$^-$CD11b$^+$F4/80$^+$; N.S. n = 6). Unpaired *t* test.

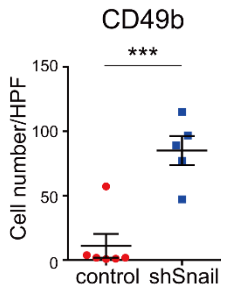

Supplementary Figure 5. NK infiltration did not affect tumor progression in mouse tumors.

Immunostained CD49b^{+} cell count in HM-1-control and HM-1-shSnail (sh1) subcutaneous tumors from immunocompetent mice. HPF, high power field; n = 5-6. ***P < 0.001. Unpaired *t* test.

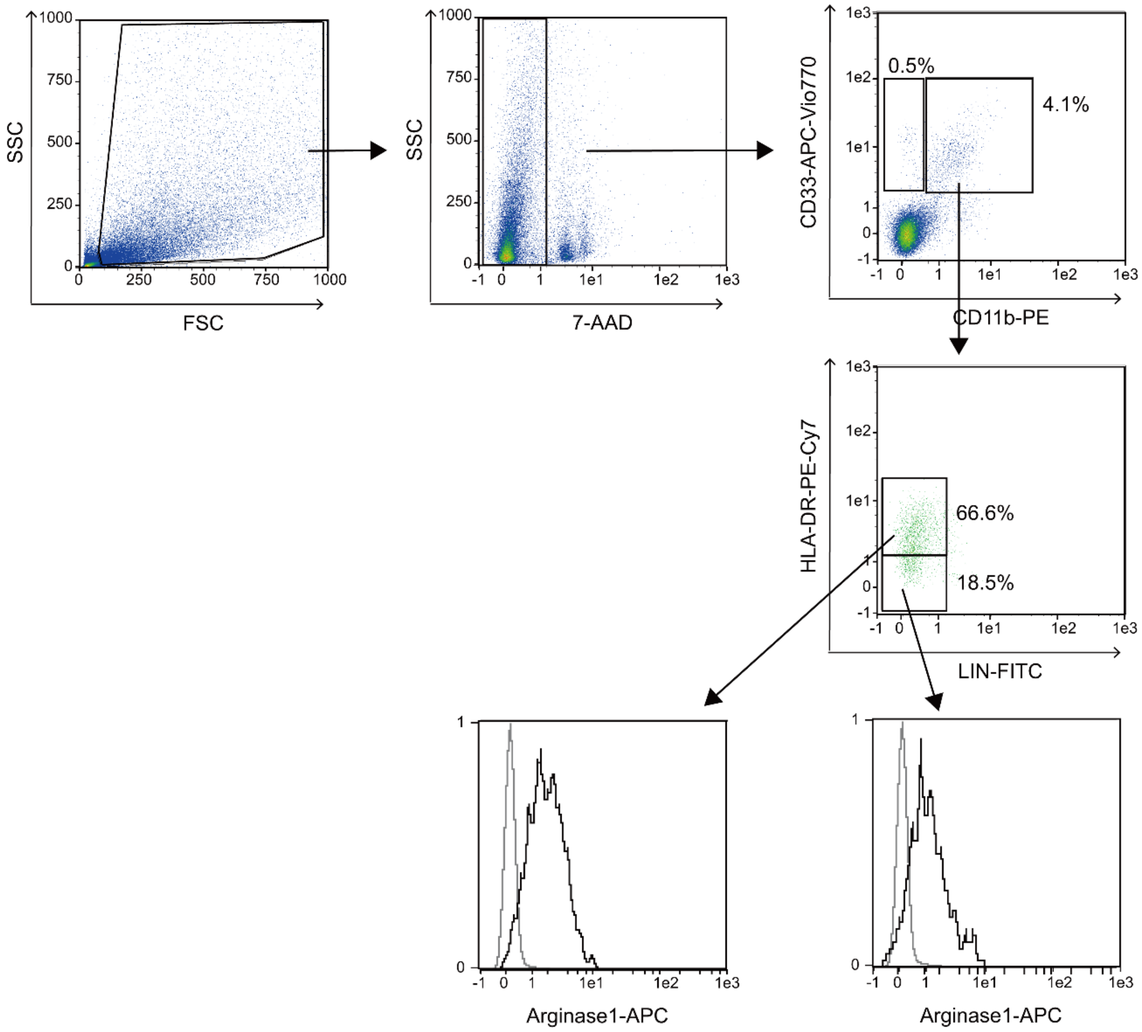

Supplementary Figure 6. CD33⁺ cells from a human ovarian cancer sample exhibit Arginase 1 expression.

Flow cytometric analyses of Arginase 1 expression in human ovarian cancer sample data. 7-AAD, $CD11b^+$, and $CD33^+$ cells were gated and analyzed for Arginase 1 expression. The gray line shows the isotype control; the black line shows Arginase 1^+ cells. The percentage of gated cells to total cells is indicated in CD33/CD11b panel (upper right) and the percentage of gated cells to $CD33^+$ cells is indicated in HLA-DR/LIN panel (middle).

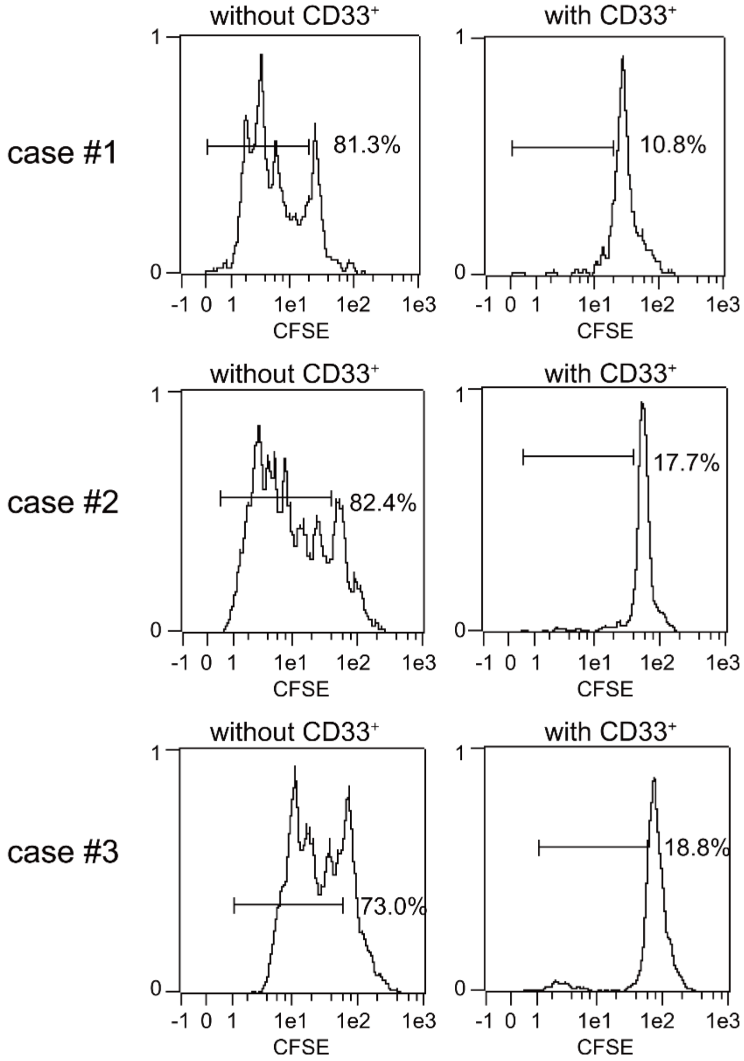

Supplementary Figure 7. CD33^{+} cells from a human ovarian cancer sample inhibit T cell proliferation.

Flow cytometric analysis of the carboxyfluorescein succinimidyl ester (CFSE)-labeled human T cell proliferation activated with Dynabeads Human T-activator CD3/28, 96 h after co-culture with or without CD33^{+} cells from human ovarian cancer samples. The ratio of CD33^{+}:CD8^{+}T cells was 1:1. The percentage of proliferating CD8^{+}T cells is indicated in each panel.

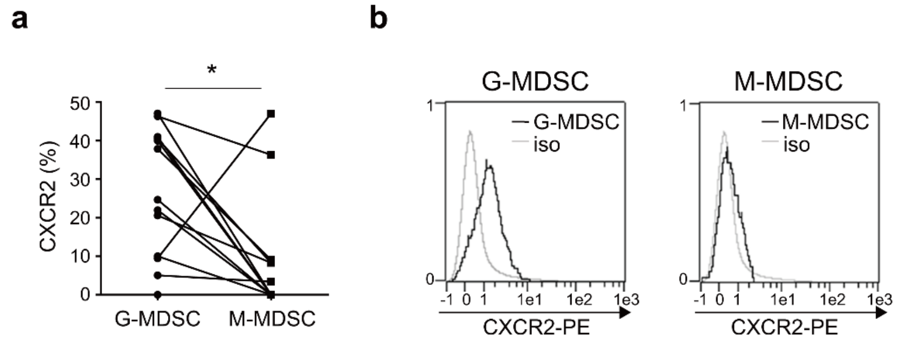

Supplementary Figure 8. CXCR2 is highly expressed on granulocytic myeloid-derived suppressor cells (G-MDSC) in ascites fluid from ovarian cancer patients.

(a) The percentage of $CXCR2^+$ cells in the ascites fluid of ovarian cancer patients; n = 13. A paired t-test was performed to calculate P values. *P < 0.05. **(b)** Representative histogram for CXCR2 expression in G-MDSC and M-MDSC. The gray line shows the isotype control; the black line shows CXCR2+ cells.

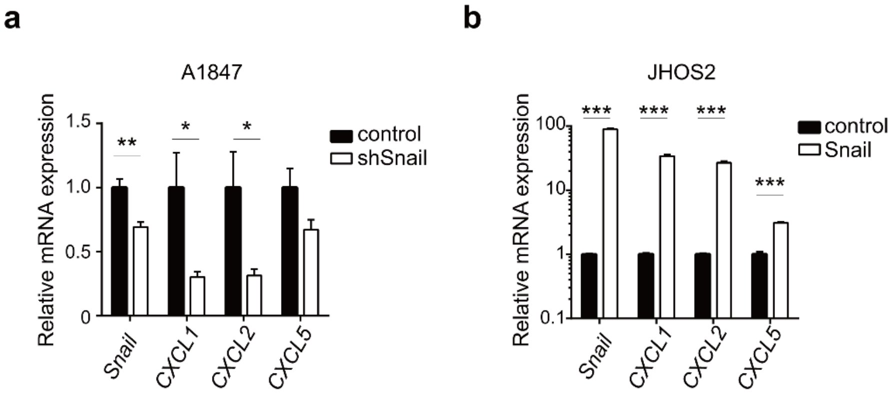

Supplementary Figure 9. Snail increases CXCR2 ligands expression in human ovarian cancer cell lines.

(a and b) Reverse transcription polymerase chain reaction (RT-PCR) of human ovarian cancer cell lines, A1847 and A1847-shSnail **(a)** and JHOS2 and JHOS2-Snail **(b)**; n = 6. *P < 0.05, **P < 0.01 and ***P < 0.001 by unpaired *t* test.

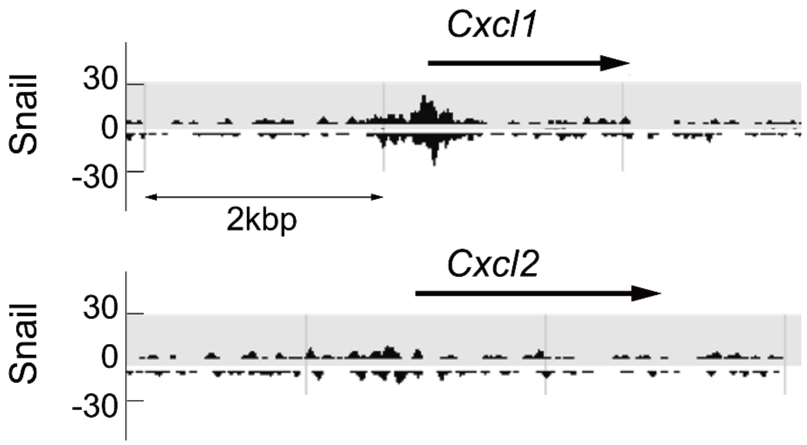

Supplementary Figure 10. Chromatin immunoprecipitation (ChIP) sequence data show that Snail might directly bind to *Cxcl1* and *Cxcl2* proximal promoters.
ChIP sequencing (ChIP-seq) signals for Snail at the *Cxcl1* and *Cxcl2* loci.

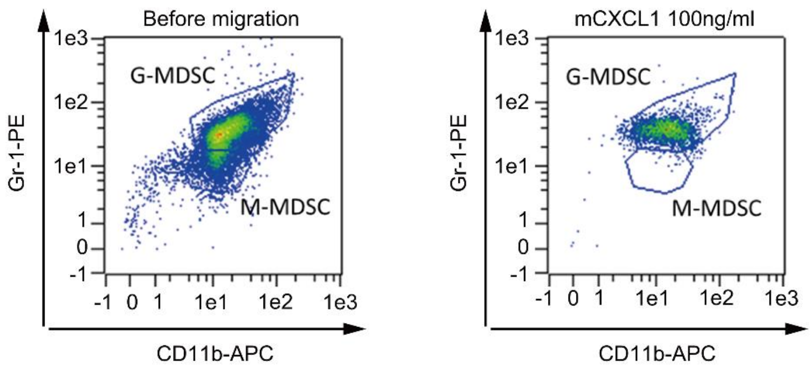

Supplementary Figure 11. CXCR2 ligands attract granulocytic myeloid-derived suppressor cells (G-MDSCs) to the tumor via CXCR2.

Flow cytometric analyses of chemotaxis assay data from MDSCs before migration and after the exposure of CXCL1 at 100 ng/mL.

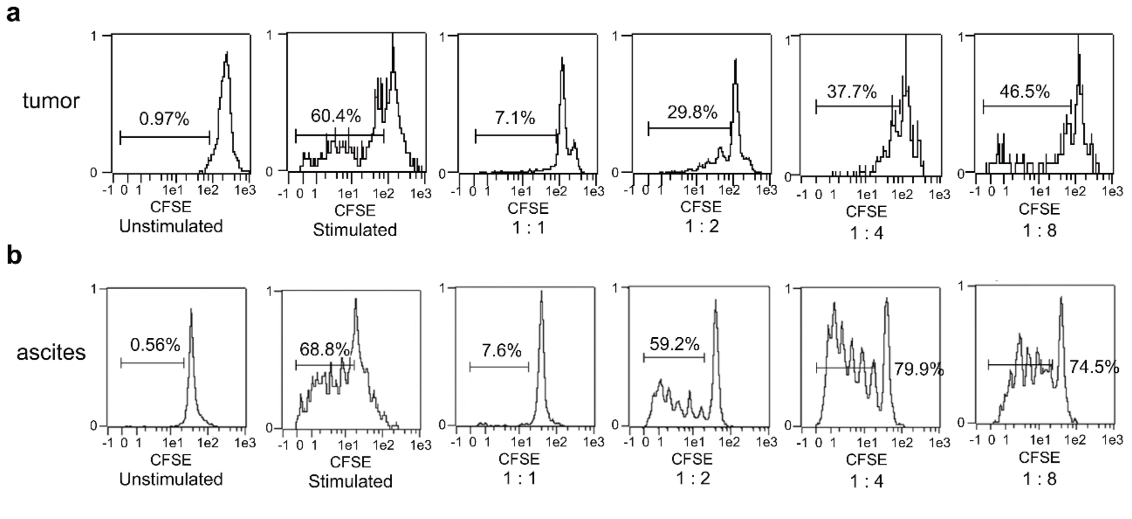

Supplementary Figure 12. MDSCs inhibit T cell proliferation.

(a and b) Flow cytometric analysis of the carboxyfluorescein succinimidyl ester (CFSE)-labeled T cell proliferation activated with Dynabeads Mouse T-activator CD3/28, 72 h after co-culture with MDSCs isolated from the subcutaneous tumors **(a)** or the ascites **(b)** of HM-1 tumor-bearing mice. The ratio means the MDSC:$CD8^{+}$T cell. The percentage of proliferating $CD8^{+}$T cells is indicated in each panel.

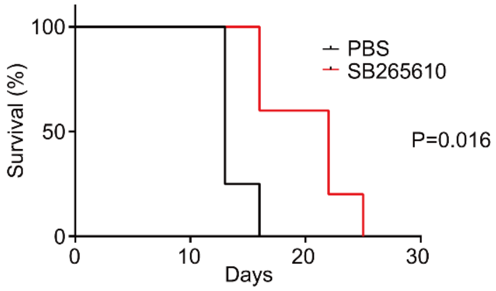

Supplementary Figure 13. A CXCR2 antagonist SB265610 suppresses ovarian tumor progression.

Survival curves for HM-1-control intraperitoneally inoculated mice treated with SB265610 or PBS six times a week from day 1 after tumor inoculation; n = 4; P = 0.016 by log-rank test.

a

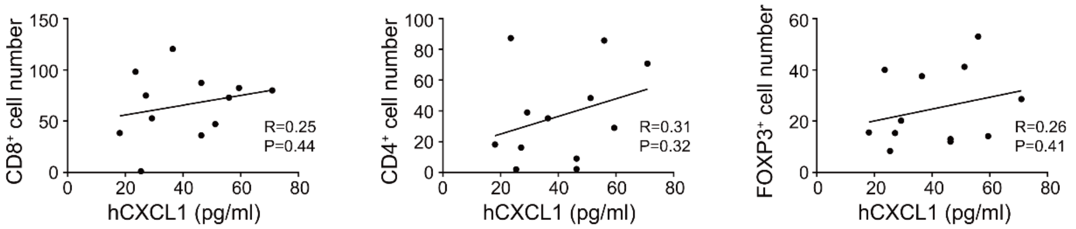

b

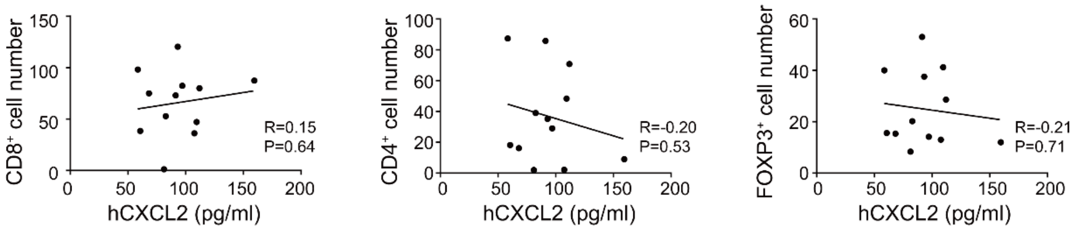

Supplementary Figure 14. Serum CXCL1 and CXCL2 levels do are not correlated with intratumoral $CD8^+$, $CD4^+$ or $FOXP3^+$ cells.

(a) Correlation between serum CXCL1 level and infiltration of $CD8^+$ (left; P = 0.44), $CD4^+$ (middle; P = 0.32) and $FOXP3^+$ (right; P = 0.41) cells in peritoneal disseminations; n = 12; Pearson's product-moment correlation analysis. **(b)** Correlation between serum CXCL2 level and infiltration of $CD8^+$ (left; P = 0.64), $CD4^+$ (middle; P = 0.53) and $FOXP3^+$ (right; P = 0.71) cells in peritoneal dissemination; n = 12; Pearson's product-moment correlation analysis.

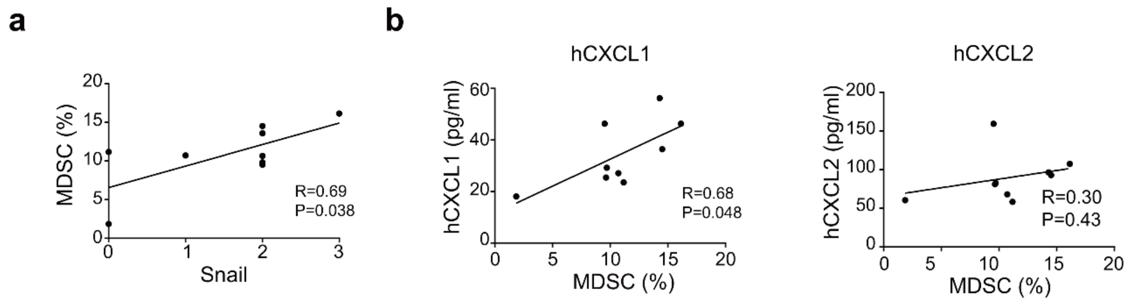

Supplementary Figure 15. MDSCs in PBMCs are associated with Snail expression in ovarian cancer patients.

(a) Correlation between Snail staining scores and MDSCs in PBMCs of ovarian cancer patients (n = 9); P = 0.038, R = 0.69. Pearson’s product-moment correlation analysis. **(b)** Correlation between serum CXCL1 (left; P = 0.048, R = 0.68) and CXCL2 (right; P = 0.43, R = 0.30) levels and MDSCs in PBMCs of ovarian cancer patients (n = 9); Pearson’s product-moment correlation analysis.

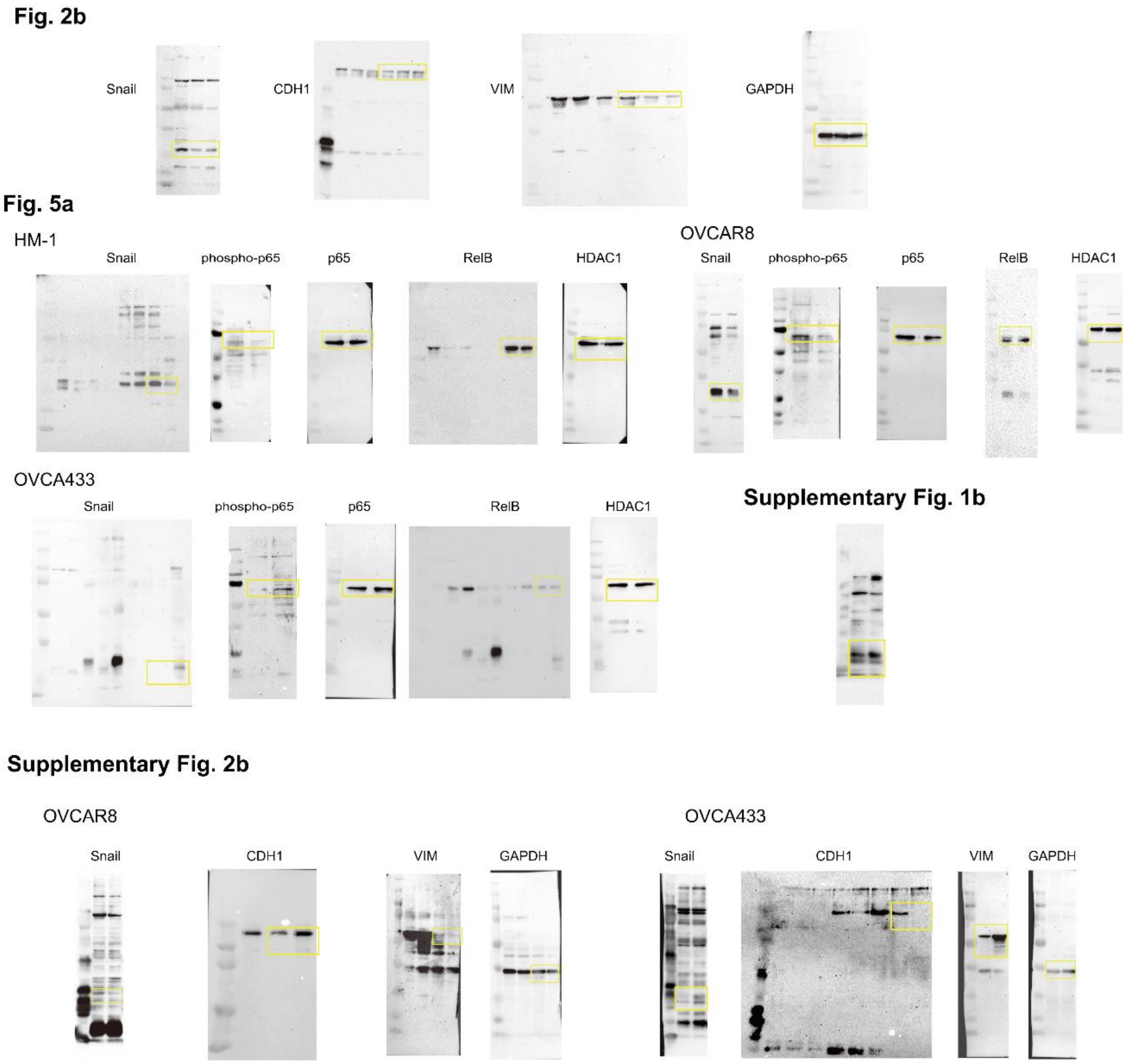

Supplementary Figure 16. Raw data of immunoblotting.

	Univariate			Multivariate		
	RR	95%CI	P value	RR	95%CI	P value
Age ≥ 55	2.22	1.03-4.45	0.045*	1.86	0.79-4.39	0.15
FIGO stage*	1.86	0.73-6.70	0.17	0.37	0.076-1.77	0.21
Distant metastasis	2.62	1.27-13.16	0.020*	4.84	1.01-23.18	0.048*
Residual tumor	2.85	1.60-8.68	0.0027*	1.95	0.78-4.86	0.15
Snail expression	2.58	1.16-5.00	0.021*	2.79	1.10-7.05	0.031*

*statistical significance

Supplementary Table 1. Multivariate analysis of prognostic factors associated with overall survival.

*FIGO stage: FIGO stage IV vs III. RR, relative risk; CI, confidence interval.

Transcript Cluster ID	Fold Change (linear)	ANOVA p-value	Gene Symbol	Description
**TC1100001219.mm.1**	-66.3	2.55E-08	Ccl2	chemokine (C-C motif) ligand 2
**TC0500000841.mm.1**	-17.36	0.000018	Cxcl5	chemokine (C-X-C motif) ligand 5
**TC0500000850.mm.1**	-13.15	0.000009	Cxcl2	chemokine (C-X-C motif) ligand 2
**TC0300002068.mm.1**	-10.43	4.90E-07	Gpr149	G protein-coupled receptor 149
**TC0100001495.mm.1**	-9.01	0.000009	Sele	selectin, endothelial cell
**TC1600001570.mm.1**	-5.84	4.66E-09	Gm23306	predicted gene, 23306
**TC1500000409.mm.1**	-5.36	0.000004	Wdyhv1	WDYHV motif containing 1
**TC1100001220.mm.1**	-5.33	0.000062	Ccl7	chemokine (C-C motif) ligand 7
**TC0700004009.mm.1**	-4.98	0.000025	Gm8995, RP23-465 M17.1	predicted gene 8995, GTPase, very large interferon inducible 1 (Gvin1) pseudogene
**TC1600001742.mm.1**	-4.93	0.000003	Nfkbiz	nuclear factor of kappa light polypeptide gene enhancer in B cells inhibitor, zeta
**TC0100003057.mm.1**	-4.89	0.000001	Ikbke	inhibitor of kappaB kinase epsilon
**TC1400001012.mm.1**	-4.84	0.00001	Gfra2	glial cell line derived neurotrophic factor family receptor alpha 2
**TC1700002352.mm.1**	-4.58	0.000011	C3	complement component 3
**TC0300000137.mm.1**	-4.45	0.000032	Cp	ceruloplasmin
**TC0700001385.mm.1**	-4.21	0.000004	Gdpd5	glycerophosphodiester phosphodiesterase domain containing 5
**TC0500002685.mm.1**	-4.03	0.000073	Tmprss11 b, Tmprss11 bnl	transmembrane protease, serine 11B, transmembrane protease, serine 11b N terminal like
**TC1300002715.mm.1**	-3.95	0.000158	Itga2	integrin alpha 2
**TC0100002177.mm.1**	-3.91	0.000031	Neurl3	neuralized homolog 3 homolog (Drosophila)
**TC1400000421.mm.1**	-3.89	2.07E-07	1700024B 05Rik	RIKEN cDNA 1700024B05 gene, novel DUF622 domain containing

				protein (1700024B05Rik)
**TC1100000475.mm.1**	-3.89	0.000097	Havcr2	hepatitis A virus cellular receptor 2
**TC1200001768.mm.1**	-3.69	0.000078	Nfkbia	nuclear factor of kappa light polypeptide gene enhancer in B cells inhibitor, alpha
**TC1500001570.mm.1**	-3.65	0.000004	Fbxo32	F-box protein 32
**TC0100000718.mm.1**	-3.64	0.000219	Ccl20	chemokine (C-C motif) ligand 20
**TC1400000430.mm.1**	-3.58	0.000005	Gm10378	predicted gene 10378, Novel DUF622 domain containing protein
**TC1700000703.mm.1**	-3.49	0.000017	Ier3	immediate early response 3
**TC0500000844.mm.1**	-3.35	0.000062	Cxcl3	chemokine (C-X-C motif) ligand 3
**TC1400000425.mm.1**	-3.2	0.000003	Gm7995	predicted gene 7995, Novel DUF622 domain containing protein
**TC0400003522.mm.1**	-3.1	0.000097	Zc3h12a	zinc finger CCCH type containing 12A
**TC0200003303.mm.1**	-3.08	0.000489	Lcn2	lipocalin 2
**TC1100000776.mm.1**	-3.06	0.000008	Aldh3a1	aldehyde dehydrogenase family 3, subfamily A1
**TC0400000458.mm.1**	-3.06	0.00025	Glipr2	GLI pathogenesis-related 2
**TC1400000423.mm.1**	-2.99	0.000245	Gm7991	predicted gene 7991, Novel DUF622 domain containing protein pseudogene
**TC1400000419.mm.1**	-2.96	0.000074	Gm8065	predicted gene 8065, Novel DUF622 domain containing protein pseudogene
**TC1400000420.mm.1**	-2.95	0.000002	1700049E17Rik1	RIKEN cDNA 1700049E17 gene, gene 1
**TC1400000426.mm.1**	-2.94	0.000264	Gm2959	predicted gene 2959, Novel DUF622 domain containing protein pseudogene
**TC0500002687.mm.1**	-2.84	0.000123	Tmprss11e	transmembrane protease, serine 11e
**TC0200004537.mm.1**	-2.8	0.000083	Atp8b4	ATPase, class I, type 8B, member 4
**TC0600000764.mm.1**	-2.79	0.000262	Tnip3	TNFAIP3 interacting protein 3
**TC1400000431.mm.1**	-2.73	0.000102	Gm3008	predicted gene 3008, Novel DUF622 domain containing protein

				pseudogene
**TC1100003097.mm.1**	-2.71	3.84E-08	Cxcl16	chemokine (C-X-C motif) ligand 16
**TC1100002176.mm.1**	-2.69	0.000033	Myo1g	myosin IG, myosin 1G
**TC1000001813.mm.1**	-2.69	0.000201	Tnfaip3	tumor necrosis factor, alpha-induced protein 3
**TC0700004171.mm.1**	-2.67	0.000093	Syt17	synaptotagmin XVII
**TC1400000432.mm.1**	-2.66	0.000101	Gm7152	predicted gene 7152, Novel DUF622 domain containing protein pseudogene
**TC1900000497.mm.1**	-2.66	0.000115	Fas	Fas (TNF receptor superfamily member 6)
**TC1100002770.mm.1**	-2.65	0.000037	Tnip1	TNFAIP3 interacting protein 1
**TC0200002542.mm.1**	-2.65	0.000497	Mmp9	matrix metallopeptidase 9
**TC0100001568.mm.1**	-2.61	0.00005	Sh2d1b1	SH2 domain protein 1B1
**TC0700001192.mm.1**	-2.59	0.000108	Mex3b	mex3 homolog B (C. elegans)
**TC0400002694.mm.1**	-2.57	0.000096	Klf4	Kruppel-like factor 4 (gut)
**TC1400000441.mm.1**	-2.56	0.000064	LOC101056186, Gm9732	uncharacterized LOC101056186, Novel DUF622 domain containing protein
**TC1400000435.mm.1**	-2.49	0.000107	Gm8104	predicted gene 8104, Novel DUF622 domain containing protein
**TC1400001883.mm.1**	-2.48	0.000082	Gm3371	predicted gene 3371, Novel DUF622 domain containing protein
**TC1400000444.mm.1**	-2.46	0.00009	Gm8046	predicted gene 8046, Novel DUF622 domain containing protein pseudogene
**TC0700002622.mm.1**	-2.45	0.000277	RP24-113D21.1	N/A, novel transcript
**TC1100000872.mm.1**	-2.44	0.000029	Pik3r5	phosphoinositide-3-kinase, regulatory subunit 5, p101
**TC1100000119.mm.1**	-2.43	0.000073	Upp1	uridine phosphorylase 1
**TC0300000092.mm.1**	-2.43	0.000199	Car13	carbonic anhydrase 13
**TC0300002718.mm.1**	-2.43	0.000282	Csf1	colony stimulating factor 1 (macrophage)
**TC1500001783.mm.1**	-2.39	0.000093	Slc39a4	solute carrier family 39 (zinc

				transporter), member 4
**TC1400000429.mm.1**	-2.38	0.000018	Gm8032	predicted gene 8032, Novel DUF622 domain containing protein pseudogene
**TC0200004159.mm.1**	-2.36	0.000095	Cd82	CD82 antigen
**TC0100001581.mm.1**	-2.35	0.000058	Adamts4	a disintegrin-like and metallopeptidase (reprolysin type) with thrombospondin type 1 motif, 4
**TC0X00003064.mm.1**	-2.35	0.000476	Ripply1	ripply1 homolog (zebrafish)
**TC0400001992.mm.1**	-2.3	0.000177	Tnfrsf9	tumor necrosis factor receptor superfamily, member 9
**TC1400000427.mm.1**	-2.29	0.000368	Gm8011	predicted gene 8011, Novel DUF622 domain containing protein
**TC1400001886.mm.1**	-2.25	0.000084	Gm5624	predicted gene 5624, Novel DUF622 domain containing protein
**TC1400001882.mm.1**	-2.22	0.000139	Gm5930	predicted gene 5930, Novel DUF622 domain containing protein
**TC1100000041.mm.1**	-2.21	0.000178	Lif	leukemia inhibitory factor
**TC1700001827.mm.1**	-2.17	0.000041	Sik1	salt inducible kinase 1
**TC1100000362.mm.1**	-2.17	0.000337	Lcp2	lymphocyte cytosolic protein 2
**TC1300001320.mm.1**	-2.15	0.000062	Gm25985	predicted gene, 25985
**TC1400001888.mm.1**	-2.14	0.000283	Gm8267	predicted gene 8267, Novel DUF622 domain containing protein
**TC1700000864.mm.1**	-2.06	0.000151	Nfkbie	nuclear factor of kappa light polypeptide gene enhancer in B cells inhibitor, epsilon
**TC1400000448.mm.1**	-2.06	0.000433	Gm6526	predicted gene 6526, Novel DUF622 domain containing protein
**TC0400002251.mm.1**	-2.02	0.000015	Ripk2	receptor (TNFRSF)-interacting serine-threonine kinase 2
**TC0300002482.mm.1**	-2.02	0.000142	Ecm1, Mir7014	extracellular matrix protein 1, microRNA mir-7014, mmu-mir-7014
**TC1400000415.mm.1**	-2.01	0.000117	Gm2832	predicted gene 2832, Novel DUF622 domain containing protein
**TC1500000389.mm.1**	-2.01	0.000139	Nov	nephroblastoma overexpressed gene

**TC1500000031.mm.1**	-2.01	0.000144	Dab2, LOC101055883	disabled 2, mitogen-responsive phosphoprotein, uncharacterized LOC101055883, disabled homolog 2 (Drosophila)
**TC0X00003019.mm.1**	-2	0.00018	Nxf3	nuclear RNA export factor 3

Supplementary Table 2. Downregulated (81) genes in HM-1-shSnail cells based on microarray analysis.

81 genes downregulated by more than 2-fold in Snail-knockdown cells.

Transcript Cluster ID	Fold Change (linear)	ANOVA p-value	Gene Symbol	Description
**TC1500000119.mm.1**	8.65	0.00001	Cdh9	cadherin 9
**TC0100000372.mm.1**	5.35	0.000004	Sdpr	serum deprivation response
**TC0200002945.mm.1**	3.7	0.000272	Itih2	inter-alpha trypsin inhibitor, heavy chain 2
**TC1700001178.mm.1**	3.13	0.000004	Crim1	cysteine rich transmembrane BMP regulator 1 (chordin like)
**TC1600001616.mm.1**	3.13	0.000339	Upk1b	uroplakin 1B
**TC1200001314.mm.1**	3	0.00002	Rapgef5	Rap guanine nucleotide exchange factor (GEF) 5
**TC1500000392.mm.1**	2.99	0.000009	Deptor	DEP domain containing MTOR-interacting protein, DEP domain containing 6
**TC1300002463.mm.1**	2.87	0.000032	Iqgap2	IQ motif containing GTPase activating protein 2
**TC1900000335.mm.1**	2.86	0.000005	Aldh1a1	aldehyde dehydrogenase family 1, subfamily A1
**TC0X00002872.mm.1**	2.8	0.000365	Pof1b	premature ovarian failure 1B
**TC0300003251.mm.1**	2.69	0.000305	Clca1	chloride channel calcium activated 1
**TC0400002337.mm.1**	2.56	0.000001	Gm11902	predicted gene 11902, DEP domain containing 6 (Depdc6) pseudogene
**TC1700000829.mm.1**	2.33	0.000013	Tnfrsf21	tumor necrosis factor receptor superfamily, member 21
**TC0100003422.mm.1**	2.3	0.000034	Atp1b1	ATPase, Na+/K+ transporting, beta 1 polypeptide
**TC0100002470.mm.1**	2.29	0.000025	Fzd5	frizzled homolog 5 (Drosophila)
**TC0500000860.mm.1**	2.13	0.000071	Parm1	prostate androgen-regulated mucin-like protein 1
**TC1800001234.mm.1**	2.12	0.000282	Spry4	sprouty homolog 4 (Drosophila)
**TC1900000531.mm.1**	2.07	0.000107	Hhex	hematopoietically expressed homeobox

**TC0900001561.mm.1**	2.05	0.000349	Eomes	eomesodermin homolog (Xenopus laevis)

Supplementary Table 3. Upregulated (19) genes in HM-1-shSnail cells based on microarray analysis.

19 genes upregulated by more than 2-fold in Snail-knockdown cells.

gene(mouse)	primer sequence
Snail	Fw: 5' - cttgtgtctgcacgacctgt - 3'
	Rv: 5' - aggagaatggcttctcacca - 3'
Cxcl1	Fw: 5' - gactccagccacactccaac - 3'
	Rv: 5' - tgacagcgcagctcattg - 3'
Cxcl2	Fw: 5' - cagaaaatcatccaaaagatactgaa - 3'
	Rv: 5' - ctttggttcttccgttgagg - 3'
Cxcl5	Fw: 5' – tagagccccaatctccacac - 3'
	Rv: 5' - gagctggaggctcattgtg - 3'
Gapdh	Fw: 5' - gggttcctataaatacggactgc - 3'
	Rv: 5' - ccattttgtctacgggacga - 3'

gene(human)	primer sequence
SNAIL	Fw: 5' - gctgcaggactctaatccaga - 3'
	Rv: 5' - atctccggaggtgggatg - 3'
CXCL1	Fw: 5' - tcctgcatcccccatagtta - 3'
	Rv: 5' - cttcaggaacagccaccagt - 3'
CXCL2	Fw: 5' - cccatggttaagaaaatcatcg - 3'
	Rv: 5' - cttcaggaacagccaccaat - 3'
CXCL5	Fw: 5' - ggtccttcgagctccttgt - 3'
	Rv: 5' - acgcagctctctcaacacag - 3'
GAPDH	Fw: 5' - ccccggtttctataaattgagc - 3'
	Rv: 5' - caccttccccatggtgtct - 3'

Supplementary Table 4. Primers used for real time polymerase chain reaction (RT-PCR).

antibody	clone	company
anti -mouse IFNγ	XMG1.2	BD Biosciences
anti-mouse CD8a	53-6.7	BioLegend
anti-mouse CD45	30-F11	BD Biosciences
anti-mouse Gr-1	RB6-8C5	TONBO biosciences
anti-mouse CXCR2 (CD182)	SA044G4	BioLegend
anti-mouse Ly6G	1A8	TONBO biosciences
anti-mouse Ly6C	AL-21	BD Biosciences
anti-mouse CD11b	M1/70	TONBO biosciences
Anti-mouse F4/80	BM8	BioLegend
anti -human CD11b	M1/70	TONBO biosciences
anti-human CD33	WM53	BD Biosciences
anti-human CD14	MΦ9	BD Biosciences
anti-human CD15	W6D3	BD Biosciences
anti-human CD2	RPA-2.10	BD Biosciences
anti-CD19	HIB19	BD Biosciences
anti-human CD56	B159	BD Biosciences
anti-human HLA-DR	G46-6	BD Biosciences
anti-human CXCR2 (CD182)	REA208	Miltenyi Biotec
Anti-human Arginase 1	658922	R&D Systems

Supplementary Table 5. Antibodies used for flow cytometry.
